# A Hierarchy of Luminal Transcription Factors Defines AR Cistrome and Is Lost in Neuroendocrine Prostate Cancer

**DOI:** 10.47248/chp2502020008

**Published:** 2025-05-06

**Authors:** Viriya Keo, Xiaodong Lu, Lourdes Brea, Xianglin Shi, Jindan Yu, Jonathan C. Zhao

**Affiliations:** 1 Department of Urology, Emory University School of Medicine, Atlanta, GA, USA; 2 Department of Human Genetics, Emory University School of Medicine, Atlanta, GA, USA; 3 Winship Cancer Institute, Emory University School of Medicine, Atlanta, GA, USA

**Keywords:** Neuroendocrine prostate cancer, HOXB13, FOXA1, GATA2, DNA methylation, ChIP-seq, pioneer factor, cistrome reprogramming

## Abstract

Androgen receptor (AR) is a hormonal transcription factor (TF) that binds to cis-regulatory elements of prostate lineage-specific genes to govern androgen response and progression of prostate cancer (PCa). This AR cistrome has been reported to be controlled by multiple chromatin-pioneering factors such as FOXA1, HOXB13, and GATA2. However, how these pioneer factors cooperate to regulate the AR cistrome remains unclear. Here, through comparative ChIP-seq analyses, we found that FOXA1 alone was sufficient to recruit AR to its binding sites regardless of H3K4me1. FOXA1 further enlisted HOXB13 and/or GATA2 to augment AR binding and enhancer activation, while HOXB13 and/or GATA2 alone were unable to recruit each other, nor AR. Moreover, HOXB13 knockdown attenuated AR and GATA2 expression and chromatin binding but failed to reprogram their cistromes, suggesting a role as a cofactor rather than a pioneer factor. During the neuroendocrine transformation (NET) of PCa, AR, GATA2, and HOXB13 were lost due to promoter hypermethylation, whereas FOXA1 was down-regulated by transcriptional repression. Lastly, through analyses of tissue microarrays, we confirmed that FOXA1 protein levels were drastically reduced in neuroendocrine PCa, as compared to AR-positive PCa. Therefore, our findings report a hierarchical network of TFs, pioneered by FOXA1 and facilitated by HOXB13 and GATA2, that defines lineage-specific AR cistrome and was lost during NET of PCa.

## 1. Introduction

Prostate cancer (PCa) is a leading cause of cancer-associated death in men in the United States. The androgen receptor (AR), a member of the nuclear receptor family, is required for prostate development and differentiation [1,2]. Aberrant activation of the AR signaling plays a central role in PCa initiation and progression, and AR pathway inhibitors (ARPi) are mainstay treatments for advanced PCa [1,2]. Although initially responsive to ARPi, a large number of metastatic PCa eventually recur and become castration-resistant prostate cancer (CRPC), largely due to the reactivation of AR pathways [3–5]. As a transcription factor (TF), AR controls cellular processes by binding to DNAs through the androgen response element (AREs) to directly regulate target gene expression. This AR cistrome is rigorously regulated by chromatin pioneering transcription factors, such as FOXA1, GATA2, and HOXB13 [6–9].

FOXA1 (previously known as HNF3A) is a member of the forkhead (FKHD) family TF that is highly expressed in the prostate [10,11]. As a pioneer TF, FOXA1 can access condensed chromatin to bind the FKHD motif and subsequently open up the loci for access to lineage-specific TFs [12–14]. FOXA1 preferentially binds to H3K4me1-marked lineage-specific enhancers to increase their accessibility for better recruitment of AR, competing with high-affinity ARE sites at less accessible chromatin regions. FOXA1 is thus capable of reprogramming AR binding from its ARE motif to FOXA1-bound, more accessible sites, many of which are lineage-specific enhancers [8,14–16]. FOXA1 down-regulation, as observed in CRPC [8], relinquishes AR to form strong binding at ARE sites, leading to androgen-independent oncogenic gene expression and tumor growth [8,15,16].

HOXB13, a member of homeobox-containing TF, is predominantly expressed in the prostate and, to a much lesser degree, in the colon [17,18]. In transgenic mouse models, the Hoxb13 homeobox domain deletion mutant impairs ventral prostate lobe development [19]. HOXB13 regulates AR cistrome in a context-and sequence-dependent manner. For instance, in androgen-dependent PCa cells, HOXB13 shows multifaceted roles in potentially initiating, tethering, or antagonizing AR binding to chromatin depending on ARE and Homeobox motifs in the specific genomic loci [20]. When co-expressed with FOXA1 in benign prostate cells, HOXB13 and FOXA1 reprogram AR cistrome from normal prostate- to tumor-specific binding sites [21]. In CRPC cells, such as 22Rv1 and LN95, HOXB13 co-occupied with AR-V7, a CRPC-associated AR variant, to govern AR-V7-driven oncogenic programs, including cell proliferation [22].

GATA2 is one of the six vertebrate members of the GATA family of pioneer factors, which, like FOXA1, are also capable of binding to compact chromatin and increasing the local accessibility to lineage-specific TFs [13]. GATA2 and GATA3 are highly expressed in human and mouse prostate [23]. It has been shown that GATA2 mutant mouse exhibits numerous urogenital abnormalities, such as hypoplastic seminal vesicles [24]. During prostate tumor transformation, GATA2 expression is increased and promotes PCa progression [25]. Unlike FOXA1 and HOXB13, GATA2 has been demonstrated as a positive regulator of AR cistrome and androgen response through multiple mechanisms [7,25,26]. First, GATA2 directly induces AR transcription via binding to the regulatory elements of the AR gene. Second, as a pioneer TF, GATA2 establishes an accessible local chromatin environment and facilitates AR binding to lineage-specific enhancers. Third, GATA2 recruits MED1, a submit of mediator complex, to modulate enhancer-promoter looping at AR-target genes.

While FOXA1, HOXB13, and GATA2 have each been shown to co-occupy AR binding sites and regulate AR cistrome, the extent by which each of them is individually required for AR binding to the chromatin and any hierarchy in their regulation have not been carefully investigated. We recently reported that FOXA1 not only reprograms AR but also GATA2 chromatin binding, suggesting FOXA1 acts upstream of both GATA2 and AR [7]. However, the crosstalk between HOXB13 and FOXA1/GATA2 has not been investigated. In addition, the AR program is drastically deactivated in up to 20% of CRPC, which loses AR expression and develops morphologic and molecular features of neuroendocrine (NE) PCa (NEPC). HOXB13 is highly expressed in primary PCa but becomes down-regulated in ~30% metastatic CRPC and most AR-negative PCa, including NEPC, due to promoter methylation [9,22,27,28], while FOXA1 remains to express in NEPC and is reprogrammed to mediate NE-lineage gene expression [29]. However, how these core luminal TFs, in particular GATA2, are regulated during the NE transformation of PCa has not been systematically studied.

## 2. Results

### 2.1 FOXA1 is required and sufficient in defining AR cistrome, independently of H3K4me1 and facilitated by HOXB13 and GATA2

**Figure 1:**
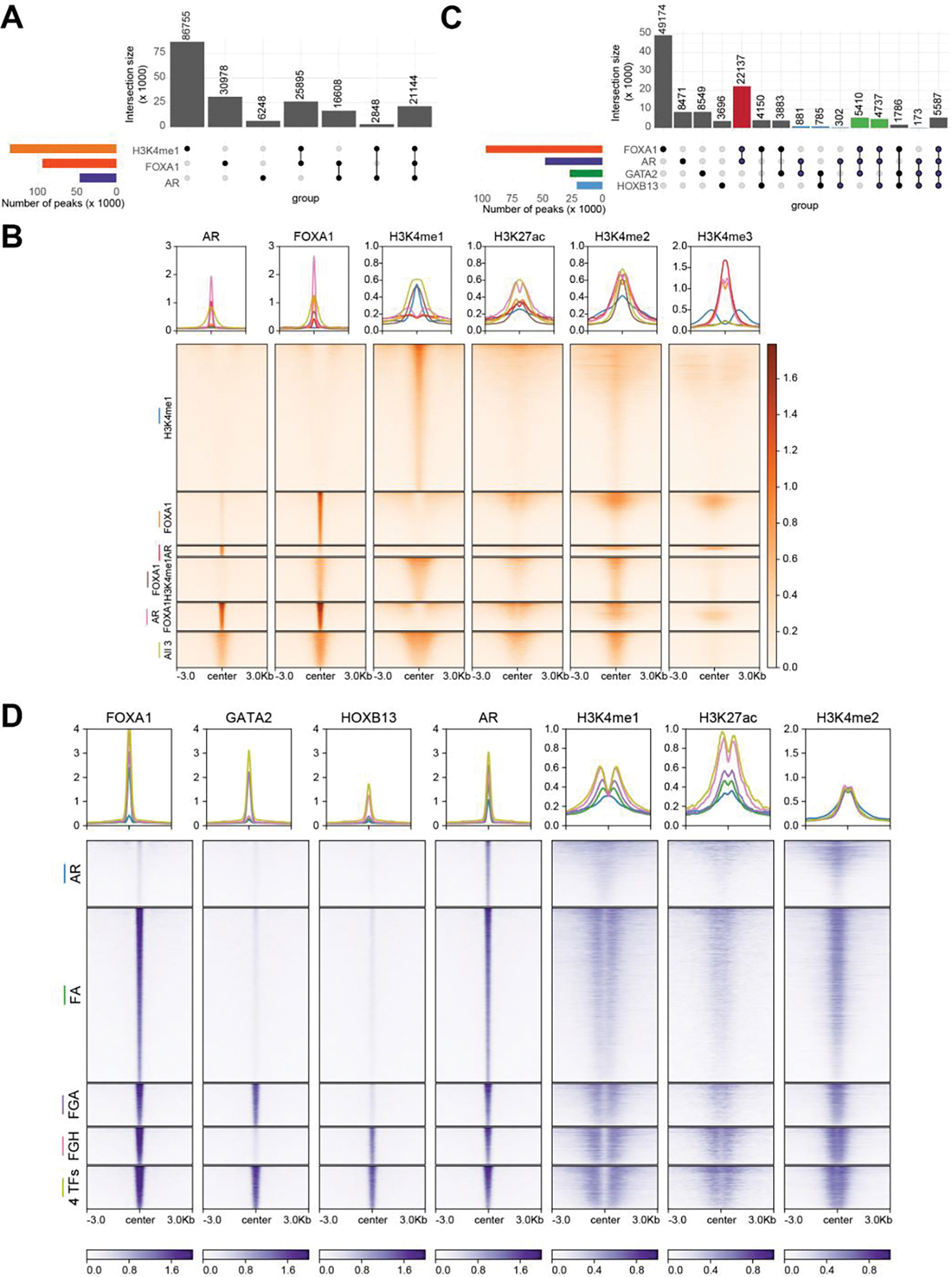
FOXA1 is required and sufficient in defining AR cistrome, independently of H3K4me1 and facilitated by HOXB13 and GATA2 (A) Upset plot showing the overlap of the binding sites of H3K4me1, AR and FOXA1 in LNCaP cells. (B) Heatmap showing binding intensities centered around ± 3kb of select H3K4me1, AR and/or FOXA1 co-bound sites. Binding of AR, FOXA1, H3K4me1, H3K27ac, H3K4me2 and H3K4me3 are shown. The color bar on the side indicates the scale of the enrichment intensity. The profile plot on top shows the averaged binding intensity across each set of binding sites. (C) Upset plot showing the overlap of the binding sites of AR, FOXA1, GATA2, and HOXB13 in LNCaP cells. Highlighted bars indicate groups of interest. Red: AR FOXA1 co-binding sites, blue: ARBS without FOXA1, green: FOXA1, AR, and either GATA2 or HOXB13 sites. (D) Heatmap showing binding intensities of AR, FOXA1, GATA2, HOXB13, H3K4me1, H3K27ac, H3K4me2 centered at ± 3kb around AR binding sites. The color bars on the bottom indicate the scale of the enrichment intensity. The profile plot on top shows the averaged binding intensity across each set of binding sites.

To decipher how AR cistrome is defined by the key pioneering factors, we performed chromatin immunoprecipitation sequencing (ChIP-seq) of FOXA1, HOXB13, GATA2, and AR, along with several histone modification markers in LNCaP cells. As H3K4me1 has been shown to be essential in guiding lineage-specific FOXA1 recruitment to the chromatin, we first evaluated the overlaps between H3K4me1, FOXA1, and AR binding (Figure 1A). Not surprisingly, there were substantially more chromatin regions with H3K4me1 mark than FOXA1 binding sites (FXBS) – only about a quarter of H3K4me1-marked enhancers were co-occupied by FOXA1, and about half of these were also bound by AR. FOXA1-co-occupied H3K4me1 sites exhibited more vigorous intensity with broader peaks and stronger H3K27ac, which were further augmented by the co-recruitment of AR, indicating that the binding of FOXA1 and, in particular AR, led to enhancer activation (Figure 1B). On the other side, there was also a large number (46%) of FXBS without H3K4me1, a third of which, surprisingly, showed very strong AR binding, and the remaining two-thirds also exhibited some basal recruitment of AR. Accordingly, focusing on the AR binding sites (ARBS), we found that over 80% of ARBS were co-occupied by FOXA1, 44% of which, however, did not contain H3K4me1 (Figure 1A). And yet, they showed comparable levels of enhancer activation, as demarcated by H3K27ac. These results support that FOXA1 alone is able to recruit AR, independently of H3K4me1, and mediate H3K27ac. In contrast, H3K4me1-only sites (without FOXA1) were completely devoid of AR, indicating its insufficiency in recruiting AR. We did notice that FOXA1/AR-only binding sites (without H3K4me1) contained higher H3K4me3, suggesting they were promoters.

In summary, our data indicates that FOXA1 is required and sufficient in recruiting AR to target chromatin to activate enhancers/promoters, and this function is largely independent of H3K4me1, albeit over 50% of FXBS are marked by H3K4me1. Next, we thought to delineate how the various pioneer factors regulate the AR cistrome. We found that FOXA1 had the highest number of binding sites, followed by AR, GATA2, and HOXB13 (Figure 1C). More than 70% of GATA-binding sites (GTBS) or HOXB13 binding sites (HXBS) are bound by FOXA1, a majority (over 60%) of which are also bound by AR, being consistent with them being cofactors. Critically, we observed that very few GATA2- or HOXB13-only (without FOXA1) sites were bound by AR, suggesting that FOXA1 is instrumental in mediating GATA2 and/or HOXB13 co-occupancy at ARBS. Likewise, GATA2 and HOXB13 barely overlapped with each other at genomic regions without FOXA1 binding, indicating that they belong to separate protein complexes. Focusing on ARBS, we observed that FOXA1 alone is sufficient to mediate more than 50% of ARBS with corresponding enhancer priming and activation, as indicated by H3K4me1 and H3K27ac (Figure 1D). GATA2 and/or HOXB13 co-bound at over 30% of ARBS, further augmenting enhancer activation. There were some solo AR binding events, which were, in general, very weak and insufficient to activate enhancers. Taken together, these results support that FOXA1 is fundamental in recruiting AR to the chromatin, and this is further enhanced by GATA2 and/or HOXB13, which, by themselves, are insufficient to recruit AR.

### 2.2 HOXB13 knockdown decreases AR and GATA2 protein levels without reprogramming their cistromes

**Figure 2:**
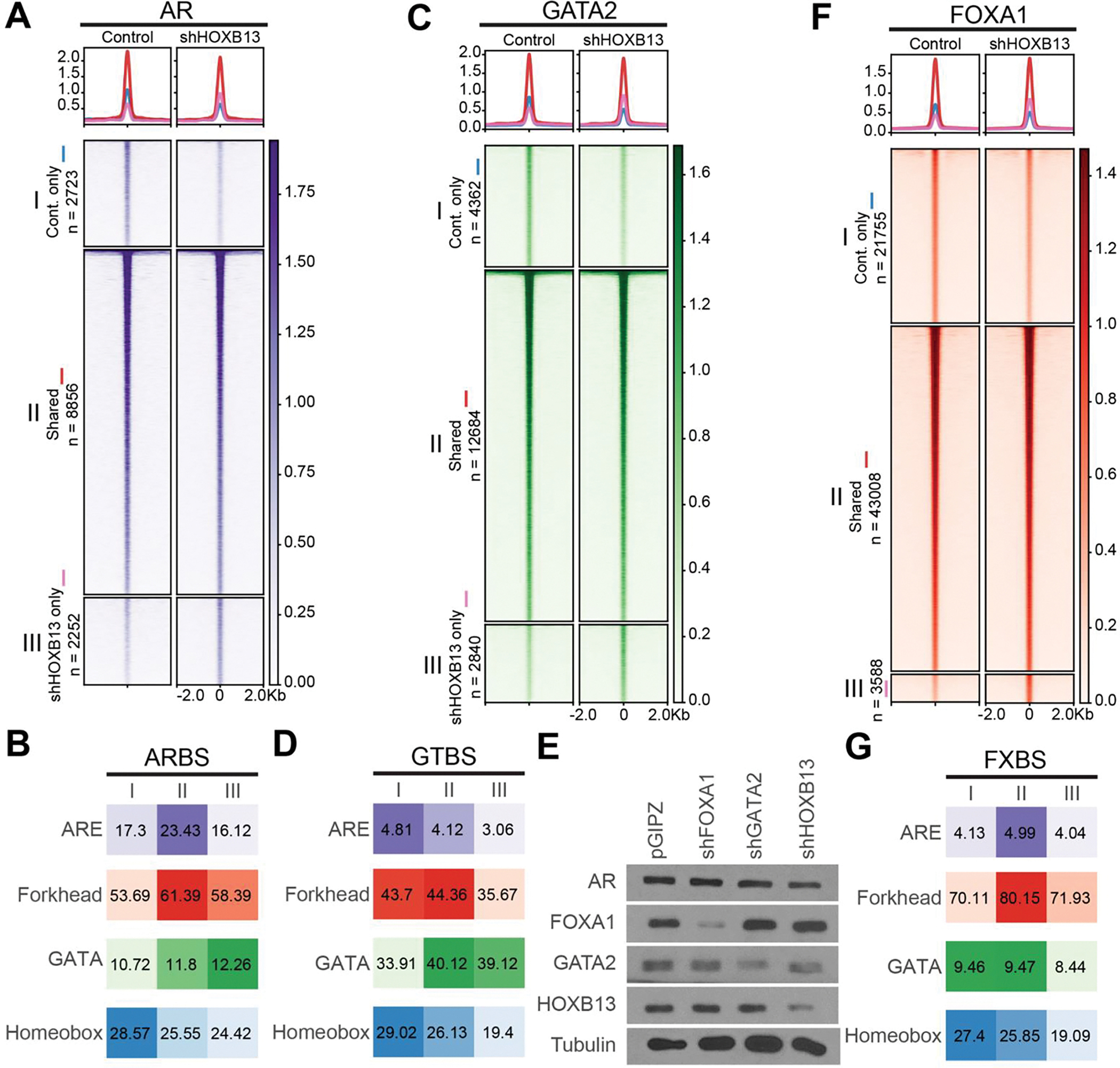
HOXB13 decreases AR and GATA2 protein levels without reprogramming their cistromes. (A, C, F) Heatmaps showing HOXB13 knockdown leads to few reprogrammed AR, GATA2, FOXA1 sites. I: control only, II: shared, III: knockdown only. The color bar on the side indicates the scale of the enrichment intensity. The profile plot on top shows averaged binding intensity across each set of binding sites. (B, D, G) Enrichment of each motif of each TF (B. AR, D. GATA2, G. HOXB13) in each category in the above heatmaps. I: control only, II: shared, III: knockdown only. Numbers indicate % of target sequences with motif found. (E) Western blot of FOXA1, GATA2 and HOXB13 KD efficiency in LNCaP cells.

Our data thus far suggest a hierarchy of TF regulation where FOXA1 functions as a pioneer factor of AR, GATA2, and HOXB13, which, however, by themselves, do not directly reprogram each other’s cistrome. We have previously reported that GATA2 is unable to reprogram FOXA1 or AR cistrome, supporting its not being a pioneer factor, while FOXA1 reprograms AR and GATA2 acting as a pioneer factor [7]. To determine how HOXB13 regulates the other TFs, we performed HOXB13 knockdown (KD) using shRNA in LNCaP cells, which were then subjected to various ChIP-seq analyses. While there was a balanced gain and loss of weak ARBS that was likely caused by technical variations, we observed an overall decrease in the binding intensity of core ARBS upon HOXB13 KD (Figure 2A). These findings are in direct contrast to the markedly increased strong ARBS upon FOXA1 KD (Figure S1A). Motif analyses revealed comparable ARE enrichment in the gained *vs*. lost ARBS upon HOXB13 KD, in contrast to the greatly enhanced ARE enrichment in ARBS gained in FOXA1-KD cells (Figure 2B & S1B). Very similar patterns were observed for GATA2 cistrome in HOXB13-KD cells (Figure 2C–D), again being distinct from those upon FOXA1 KD (Figure S1C–D). We thus hypothesized that the decrease in core ARBS or GTBS upon HOXB13 KD may be due to their reduced protein levels rather than reprogramming at the loss of pioneer factor, as in the case of FOXA1. Indeed, Western Blot (WB) analyses confirmed a decreased amount of AR and GATA2 proteins in HOXB13-KD cells (Figure 2E). Of note, the FOXA1 protein level was not altered by HOXB13 KD, nor was the FOXA1 cistrome impacted by HOXB13 KD (Figure 2F–G). Therefore, contrary to FOXA1, which reprograms AR, GATA2, and HOXB13 (Figure S1), HOXB13 did not reprogram the cistromes of AR, GATA2, and FOXA1.

### 2.3 The luminal TFs were reduced during NE transformation mostly by epigenetic mechanisms

**Figure 3:**
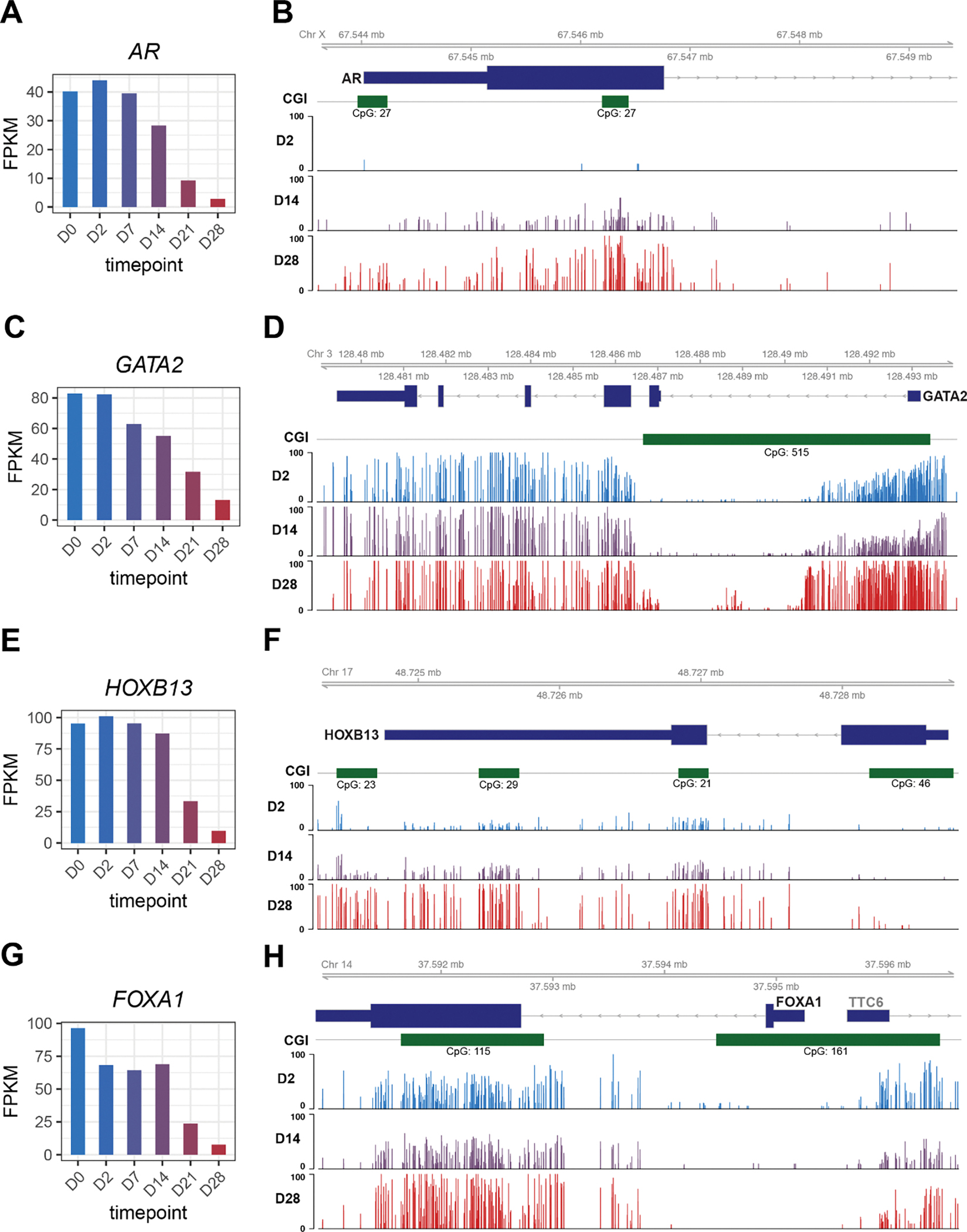
The luminal TFs were reduced during the Neuroendocrine Transformation of PCa by transcriptional or epigenetic repression. (A, C, E, G) Gene expression levels of *AR*, *FOXA1*, *GATA2*, and *HOXB13* in time-course RNA-Seq. Genes expression levels are FPKM normalized. (B, D, F, H) Gviz tracks of methylation levels of (B) *AR*, (D) *GATA2*, (F) *HOXB13* and (H) *FOXA1* from time-course RRMS. CpG islands shown in green.

We next asked what happens to these luminal TFs when PCa cells lose their luminal identities as they progress to NEPC. We utilized a NE Transformation (NET) model wherein we overexpressed FOXA2, a TF that has been associated with NEPC [30], in LNCaP cells, which underwent NET to become NEPC cells, as proven by morphology and molecular signatures, over a period of 28 days [31]. RNA-seq analyses of time-course LNCaP cells following FOXA2 overexpression revealed a gradual decrease of *AR*, starting at D7 and becoming fully depleted at D28 (Figure 3A). To gain some insights into the molecular mechanisms of its delayed-onset downregulation, we examined whole-genome DNA methylation data captured at representative D2, D14, and D28 time points and found that the AR gene promoter was devoid of methylation in LNCaP cells, being consistent with its high expression. However, it became partially methylated at D14 and fully methylated at D28, especially around the CpG island (CGI) in the promoter (Figure 3B and Figure S2A). Likewise, gradual down-regulation and promoter hypermethylation over time were also found for *GATA2* and *HOXB13* as LNCaP cells underwent NET (Figure 3C–F and Figure S2A). In contrast, FOXA1 was immediately down-regulated at D2 after FOXA2 overexpression, suggesting that it might be a direct target of FOXA2-mediated transcriptional repression (Figure 3G), which is consistent with a previous study reporting that FOXA2 significantly reduces FOXA1 expression [32]. Interestingly, a CGI approximately 2 kb downstream of the FOXA1 promoter showed some increased methylation, whereas the CGI at the promoter was not methylated in LNCaP cells and remained unmethylated throughout the NET (Figure 3H and Figure S2A). In contrast, the expression levels of NE marker genes *INSM1* and *SYP* drastically increased over the NET time course (Figure S2B–E). Of note, the valley of the CGI at the INSM1 promoter was demethylated from D0 to D28, being concordant with the gene upregulation, but the CGI shores showed increased methylation. By contrast, the *SYP* promoter was slightly demethylated and its intragenic region was hypermethylated, both of which could lead to gene upregulation, as observed for *SYP* from D0 to D28. However, these genes might also be controlled by additional mechanisms such as transcription factors. In aggregate, our data support that core luminal TFs were repressed through transcriptional or epigenetic mechanisms during NET of PCa.

### 2.4 The luminal TFs were down-regulated in Patient-Derived Xenografts (PDXs) of NEPC compared to CRPC largely due to epigenetic silencing

**Figure 4:**
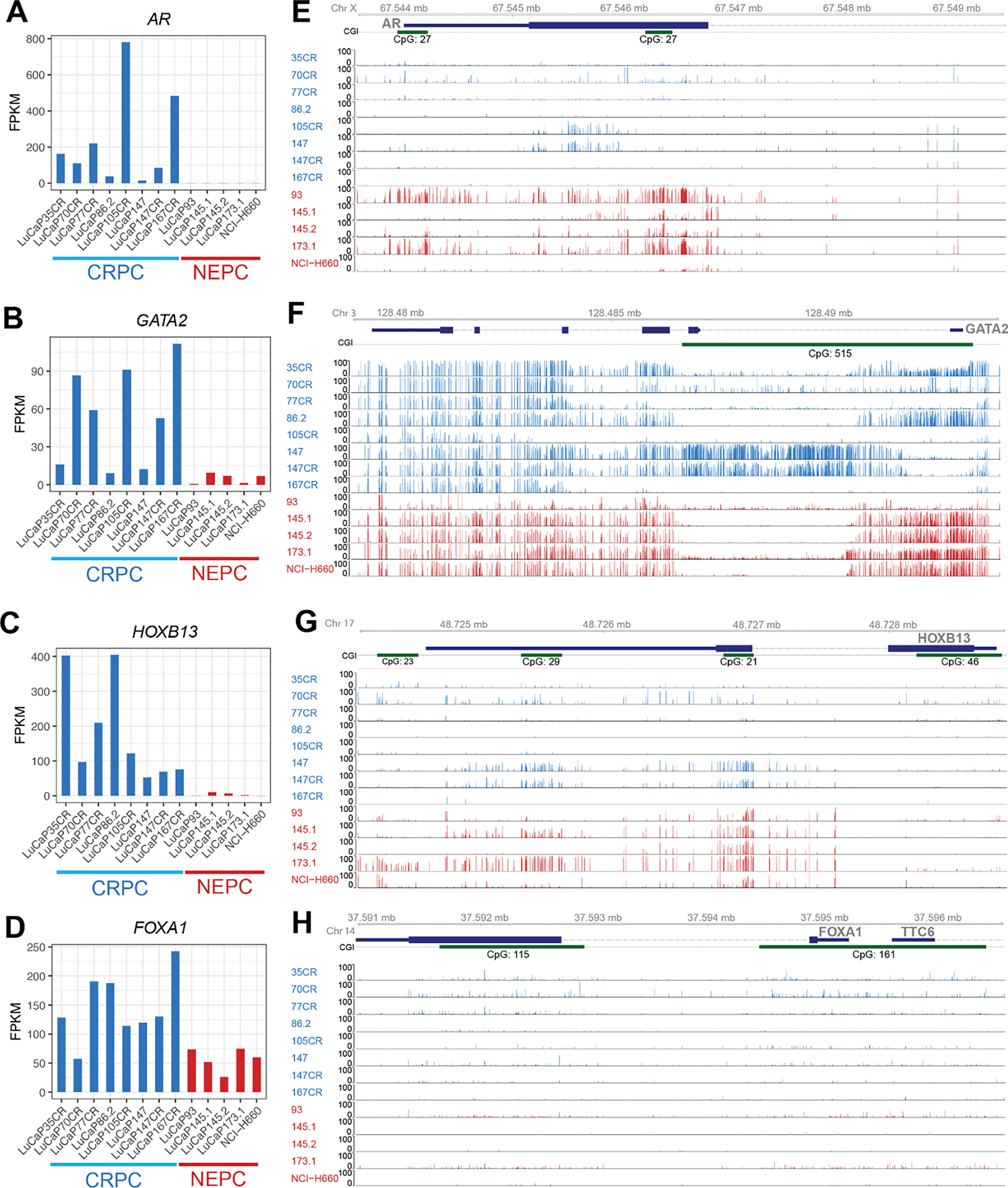
The luminal TFs were down-regulated in NEPC compared to CRPC PDXs. (A-D) Gene expression levels of *AR*, *GATA2*, *HOXB13* and *FOXA1* in CRPC (n = 8), NEPC (n = 4) PDXs and NCI-H660, a NEPC cell line. Genes expression levels are FPKM normalized. (E-H) Gviz tracks of methylation levels of (E) *AR*, (F) *GATA2* (G) *HOXB13* and (H) *FOXA1* in CRPC (blue) and NEPC PDXs and NEPC cell lines (red)s. CpG islands shown in green.

To ensure the relevance of our findings outside of our model system, we examined the expression of the four key luminal TFs in LuCaP PDX tumors. Not surprisingly, we found that *AR* mRNA level was nearly undetectable in NEPC PDXs and NEPC cell line NCI-H660, compared to CRPC PDXs (Figure 4A). Critically, *GATA2* showed some variable expression in CRPC models but was uniformly reduced in all 5 NEPC PDXs (Figure 4B). HOXB13 was abundantly expressed in all CRPC models and nearly completely lost in NEPC cells, being consistent with a recent report [9] (Figure 4C). Interestingly, *FOXA1* level was also decreased in NEPC but maintained a substantial amount of expression (Figure 4D), which agrees with its reprogrammed role to mediate the expression of neuroendocrine-lineage genes in NEPC as recently reported [29].

Next, to understand the molecular mechanisms underlying the de-regulation of these luminal TFs, we examined DNA methylation around their promoter regions utilizing previously generated whole-genome methylation data [31]. Importantly, the AR promoter regions, particularly surrounding the CGIs, harbored greatly increased DNA methylations in NEPC *vs*. CRPC PDXs (Figure 4E). Likewise, the CGI around the *GATA2* promoter was overall hypermethylated in NEPC PDXs compared to CRPC, with the exception of LuCaP93, which exhibited barely detectable GATA2 expression but also a lack of methylation (Figure 4F). Further, LuCaP147 showed a similar pattern of DNA methylation as LuCaP147CR but much less expression of *GATA2*. The lost expression of *GATA2* in these models might be caused by additional factors such as transcriptional repression. There were multiple CGIs within the *HOXB13* gene that became hypermethylated in NEPC cells (Figure 4G). On the contrary, the *FOXA1* gene lacked methylation in CRPC cells and remained hypomethylated in NEPC, suggesting that its downregulation in NEPC is likely due to other mechanisms, such as transcriptional repression (Figure 4H). Quantification of methylation levels of CGI within these genes confirmed increased *AR* and *HOXB13* methylation, variable *GATA2* methylation, and low *FOXA1* methylation in NEPC *vs*. CRPC (Figure S2F). We thus illustrated an overall downregulation of key luminal TFs during NET in NEPC compared to CRPC PDX models, largely due to epigenetic silencing.

### 2.5 The luminal TFs were reduced in clinical NEPC *vs*. CRPC tumors

**Figure 5:**
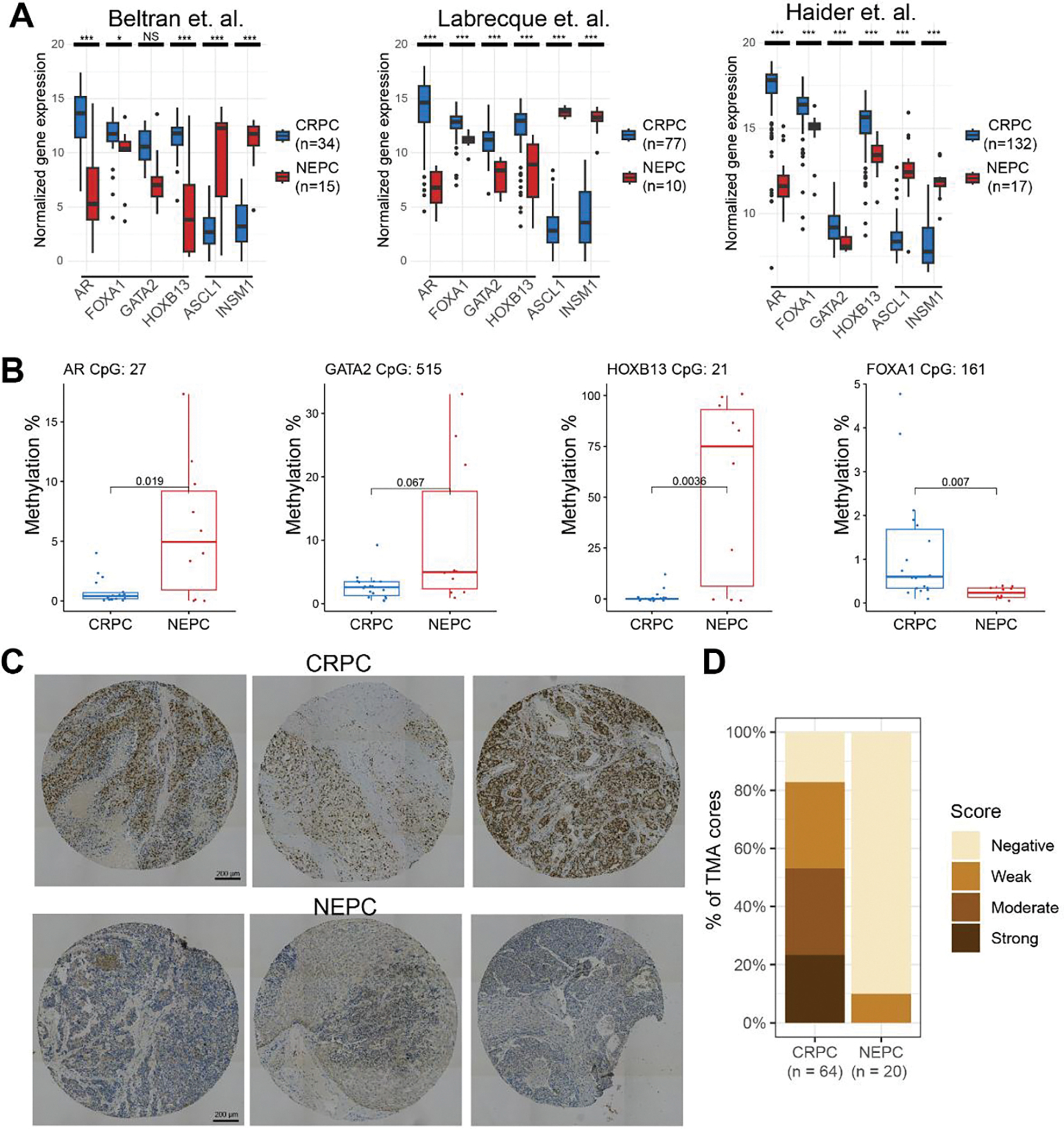
The luminal TFs were reduced in NEPC *vs*. CRPC tumors. (A) Expression of downregulated TFs (*AR*, *FOXA1*, *GATA2* and *HOXB13*) and upregulated TFs (*ASCL1* and *INSM1*) in NEPC *vs*. CRPC in 3 public datasets. Adjusted *p* values were obtained from DESeq2 for the Beltran and Labrecque (GSE126078) datasets, and from limma for the Haider (GSE74685) dataset. * *p* < 0.05, *** *p* < 0.001, NS non-significant. Log2 normalized values are shown. (B) Boxplots showing DNA methylation percentage of cytosines located in CpG islands near the promoter of each gene. *p* values were calculated using a *t-*test. CRPC n = 18, NEPC = 10 patients. (C) Representative IHC images of FOXA1 staining of CRPC and NEPC tissue cores from 3 different patients for each condition. Scale bar: 200μm. (D) Quantification of FOXA1 staining levels in NEPC (n = 20 cores) *vs*. CRPC (n = 64 cores) TMA sections. Scores are indicated as negative, weak, moderate or strong based on a binned product of the percent of positively stained cells by the staining intensity.

To confirm our findings in clinical samples, we examined three publicly available RNA-seq datasets and found that all 4 TFs are downregulated in NEPC compared with CRPC samples, while neuroendocrine TFs such as *ASCL1* and *INSM1* were up-regulated (Figure 5A). *AR* was the most strongly reduced, followed by *HOXB13*, *GATA2*, and *FOXA1*. To examine the mechanisms of their repression, we analyzed the Reduced Representation Bisulfite Sequencing (RRBS) data of 10 CRPC and 14 NEPC samples [33]. The results confirmed drastically increased DNA methylations of CGI at the *AR*, *GATA2*, and *HOXB13* promoters in NEPC compared to CRPC tumors (Figure 5B). In particular, AR and HOXB13 were barely methylated (mostly <2% methylated C) in CRPC tumors, whereas *GATA2* exhibited some low-level (mostly <5%) methylation. *AR*, *HOXB13*, and *GATA2* promoters became significantly hypermethylated in NEPC tumors, despite a few cases that remained a low level of methylation. Surprisingly, there was a clear hypomethylation of the *FOXA1* promoter in NEPC compared to CRPC in this dataset, although its expression was significantly down-regulated, further supporting that methylation is not the primary mode of regulation for FOXA1.

While *AR*, *HOXB13*, and *GATA2* were markedly downregulated or lost, with concurrent promoter hypermethylation, in various NEPC models and clinical samples that we have examined, the decrease of FOXA1 expression was less robust and lacked concordant epigenetic changes. This prompted us to examine the extent of its maintained expression in human NEPC *vs*. CRPC at the protein levels. To this end, we performed immunohistochemistry (IHC) staining of FOXA1 on tissue microarrays (TMA) containing metastatic CRPC or NEPC tissues stained positive for AR/PSA or CHGA/SYP, respectively. FOXA1 displayed strong and specific staining in the nuclei of CRPC tumors, which was reduced in many NEPC cases (Figure 5C). Quantification of FOXA1 staining for each tissue core using a scoring system based on the product of the staining intensity and the percentage of cancer cells with positive staining revealed that over 80% of CRPC tumors expressed some detectable levels of FOXA1 protein and over 50% manifested moderate to strong staining (Figure 5D). On the contrary, FOXA1 protein was not detectable in the majority of NEPC tumors – only about 10% of NEPC tumors expressed some weak levels of FOXA1 at the protein level, in contrast to the moderate decrease of FOXA1 RNA in NPEC as noted earlier.

## 3. Discussion

**Figure 6:**
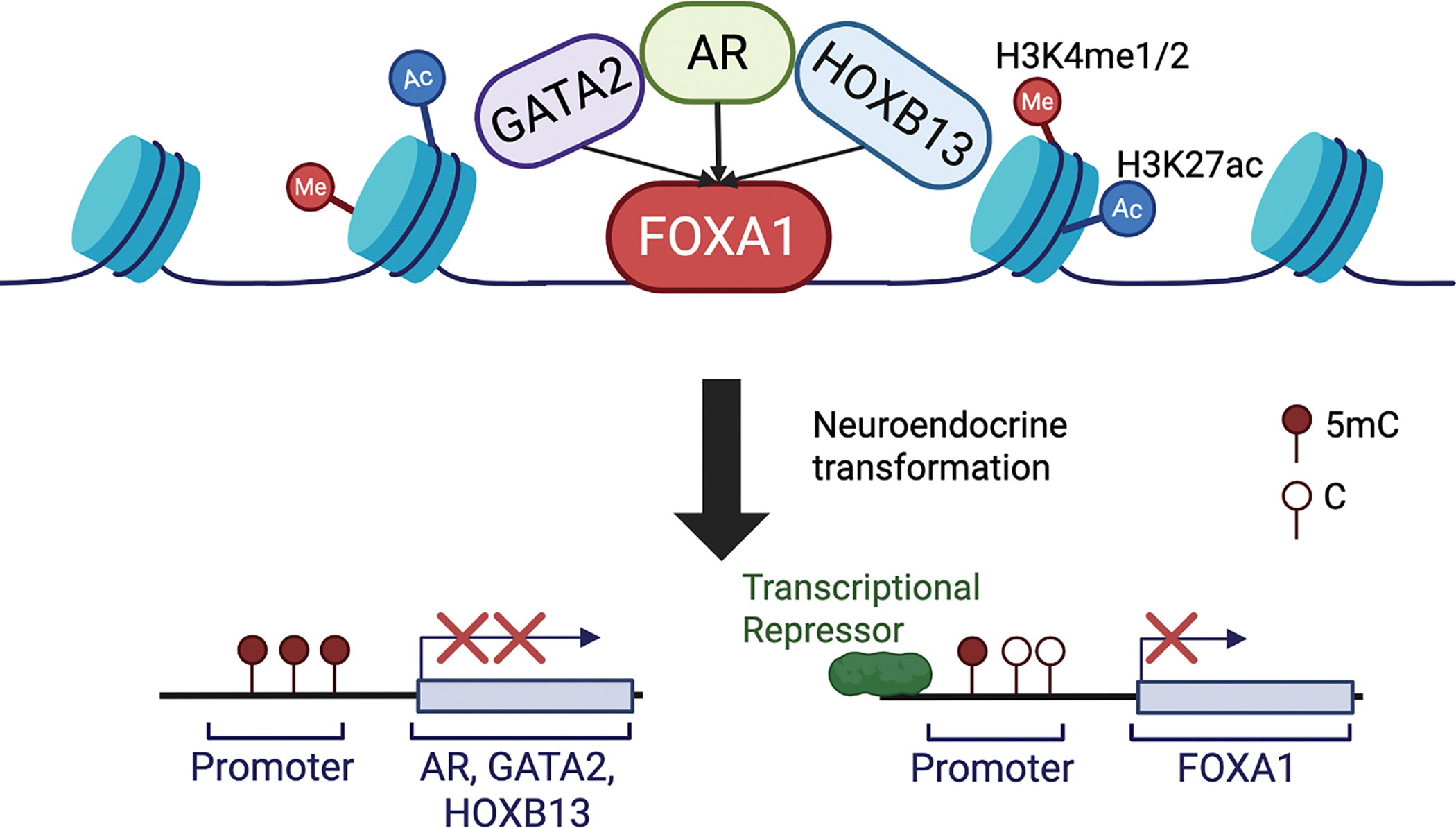
A model depicting how FOXA1 acts as a pioneer factor to recruit HOXB13 and GATA2 to tightly control AR cistrome. FOXA1 recruits AR to enhancers independently of H3K4me1. FOXA1 itself is sufficient to enlist AR to its genomic binding sites, about 60% of which lead to enhancer priming and activation. In contrast, HOXB13 and/or GATA2 alone are insufficient to recruit each other, nor AR to the target genomic sites, while AR is able to recruit HOXB13 and GATA2 to strengthen its own cistrome. During NET of PCa cells, AR, GATA2, and HOXB13 became down-regulated or lost due to promoter hypermethylation, whereas FOXA1 was decreased by transcriptional repression, dissolving AR signaling.

Previous studies have reported that FOXA1 forms distinct lineage-specific binding in different tissues, which is dependent on the distribution of H3K4me1 in the tissue types [14]. Being also a pioneer factor for AR, FOXA1 was thought to translate this cell-type-specific epigenetic signature to lineage-specific gene expression [8,14–16]. Whether FOXA1 requires the presence of H3K4me1 to recruit AR and whether H3K4me1 itself can shape AR binding, however, have not been addressed. In this study, we first confirmed that more than 50% of FOXA1 binding sites are co-occupied by H3K4me1 – 25% of total H3K4me1. Albeit it is beyond the scope of this study to determine whether the H3K4me1 mark recruits FOXA1, the data supports significant FOXA1 occupancy at lineage-specific enhancers (defined by the H3K4me1 mark). However, nearly 50% of FOXA1 binds at chromatin regions lacking H3K4me1, and surprisingly, FOXA1 alone is able to recruit AR to these sites, as opposed to H3K4me1-only sites that are often devoid of AR binding. We thus proposed a revised model wherein FOXA1 alone is necessary and sufficient to recruit AR to the chromatin (Figure 6), some of which could be promoters, while H3K4me1 helps guide the complex to lineage-specific enhancers.

FOXA1, HOXB13, and GATA2 have all been known as pioneer factors [10,11,13,34]. While FOXA1 has been shown to be crucial in reprogramming AR to lineage-specific enhancers containing FKHD motifs and bound by FOXA1, we have previously found that GATA2 is unable to reprogram AR or FOXA1 to GATA motifs [7]. Through comprehensive analyses of their binding sites, we found that FOXA1 co-occupancy is almost a prerequisite for AR to bind to the chromatin regions that were bound by GATA2 and/or HOXB13, which, by themselves, do not co-occupy with each other, nor with AR. Moreover, similar to GATA2, HOXB13 knockdown also failed to reprogram the cistromes of AR, FOXA1, or GATA2, opposing its being a pioneer factor. This agrees with the literature that provided less direct evidence for HOXB13 as a pioneer factor, either focusing on other HOX genes or only pioneering AR-V7 [22]. Only recently HOXB13 has been shown to increase accessibility through SMARCD2. We thus propose a model of hierarchical regulation wherein FOXA1 acts as a pioneer factor to bind to the chromatin to independently recruit/reprogram AR, GATA2, or HOXB13, which further facilitates/enhances the occupancy of each other on the chromatin (Figure 6).

We found that AR and HOXB13 were down-regulated during NET of PCa and in clinical NEPC *vs*. CRPC tumors associated with DNA hypermethylation, which is consistent with previous reports [9]. A previous study has reported that FOXA2, which is up-regulated in NEPC, directly suppresses FOXA1 [30], which is consistent with our data showing FOXA1 loss in our FOXA2-driven NET model. However, the RNA levels of FOXA1 were decreased but maintained in NEPC PDXs and clinical NEPC tumors, compared to CRPC, and this was not due to epigenetic silencing. Strikingly, although *FOXA1* mRNA levels were maintained in PDX and patient NEPC samples in multiple cohorts, IHC staining of TMA revealed a lack of FOXA1 protein in NEPC tumors. However, this data is limited by the small sample size – only 20 NEPC cores from 7 metastatic sites of 3 patients (1 core unreadable). Further validation of FOXA1, and perhaps GATA2 and HOXB13 proteins, in large cohorts of NEPC samples, are warranted to validate the loss of these core luminal TFs in NEPC at the protein level.

## 4. Methods

### 4.1 ChIP-seq and Western Blots

LNCaP cells were infected with shRNAs targeting control (pGIPZ), FOXA1 (shFOXA1), HOXB13 (shHOXB13) and GATA2 (shGATA2) for 5 days. Then the cells were collected and subjected to WB.

LNCaP cells were infected with control or HOXB13, FOXA1, GATA2 KD lentivirus and subjected to ChIP-seq assay as previously described [9]. Antibodies used in this study include AR (Millipore Sigma, 06–680), FOXA1 (Abcam, ab23738), HOXB13 (Santa Cruz, SC-66923), GATA2 (Santa Cruz, SC-9008), H3K4me1 (Abcam, ab8895), H3K4me2 (Millipore, 07–030), H3K27ac (Abcam, ab4729).

### 4.2 ChIP-seq analysis

ChIP-seq libraries were mapped to GRCh38 using bowtie2. Bigwig files were created using deeptool’s bamCoverage using Counts Per Million normalization with blacklisted regions removed. Peaks were called using MACS2 using -nomodel –shift 0 –extsize 250 -q 0.01 parameters. Broad peaks were called for H3K4me1. Peak overlapping analysis was performed using Homer’s mergePeaks function. This procedure produced unique and overlapping peak numbers which were provided as the intersection size to ComplexUpset to create the upset plots. Motif analysis was performed using findMotifsGenome from Homer using default parameters. Enrichment of motifs were selected from the % of Target Sequences with Motif from Homer’s known motif enrichment results. Only one motif was picked for each TF: FOXA1(Forkhead)/LNCAP-FOXA1-ChIP-Seq(GSE27824)/Homer, ARE(NR)/LNCAP-AR-ChIP-Seq(GSE27824)/Homer, Gata2(Zf)/K562-GATA2-ChIP-Seq(GSE18829)/Homer, and HOXB13(Homeobox)/ProstateTumor-HOXB13-ChIP-Seq(GSE56288)/Homer.

### 4.3 RNA-Seq analysis

NEPC time-course RNA-Seq libraries were mapped to hg38 using STAR. Output raw counts were converted into FPKM values using an in-house Perl script. FPKM values of RNA-seq data in PDXs were obtained from [35]. Public data from studies [33] and [36] were downloaded and reprocessed as above. Differential gene analysis was performed using DESeq2 between CRPC and NEPC patients in each dataset. DESeq2-normalized values were log2 transformed and plotted. Microarray data were processed using limma and log2 normalized.

### 4.4 Methylation analysis

The RRMS libraries were performed using the protocol from Oxford Nanopore. Briefly, the genomic DNA of LuCaP PDX was extracted using Quick-DNA Miniprep Plus Kit (Zymo, D4068. The DNA libraries were prepared using Ligation Sequencing Kit (ON SQK-LSK110) per the manufacturer’s protocol. Sequencing was performed on an Oxford Nanopore GridiON sequencer with R9.4.1 flow cells (FLO-MIN106D) from Oxford Nanopore. CpGs, including CpG islands, shores, shelves as well as promoter regions, were used as regions of interest for RRMS. Base calling and alignment were processed by guppy, minimap2 and remora. Visualization of genomic tracks were done using Gviz. Raw RRBS data was obtained from dbGAP phs000909.v1.p1 [33] and re-processed using Bismark. For each CpG island near the gene promoter, the methylation percentages captured for each CpG site were averaged across the entire CpG island. This was repeated for each patient and each gene of interest. *P* values between groups were calculated by t test.

### 4.5 Tissue microarrays

Tissue microarrays (TMAs) of human metastatic CRPC and NEPC specimens were obtained from the University of Washington Medical Center Prostate Cancer Donor Program. All specimens were collected from patients within 8 hours of death, formalin-fixed (decalcified in formic acid for bone specimens), paraffin-embedded, and examined histologically for the presence of a nonnecrotic tumor. TMAs were annotated as NEPC based on lack of AR, PSA, NKX3–1 and presence of CHGA and SYP IHC staining, which were scored by the University of Washington. 8 CRPC patients and 3 NEPC patients from Array C were included. Each patient has 1–3 metastatic sites, and each site has 3 cores. TMAs were stained for FOXA1 using anti-FOXA1 (1:400; ab23738, Abcam). FOXA1 staining for each TMA core was scored as previously described [37]. In brief, the percentage of positively stained tumor cells was assigned a score between 0–4, indicating ≤5%, 6%−25%, 26%−50%, 51%−75%, and 76%−100% positive cells, respectively. The staining intensity was scored between 0–3 for absent, weak, moderate and strong staining, respectively. The products of the two scores were obtained to produce final scores (between 0–12), which were then binned as follows: negative (0–1), weak (2–4), moderate (5–8), and strong (9–12).

## Supplementary Material

supplementary material Figure S1: FOXA1 reprograms the binding of AR, GATA2, and HOXB13 to many new binding sites that are enriched for their own motifs.
Figure S2: NE markers have increased expression and variable methylation over NET.

Supplementary Information

The following supplementary materials are available on the website of this paper:

## Data Availability

Some previously generated ChIP-Seq data are deposited at GSE69045. The time-course RNA-Seq and RRMS are deposited at GSE239278. All newly generated data used in this paper are deposited at GSE296178.

## Abbreviations

The following abbreviations are used in this manuscript:

PCa: Prostate cancer
NEPC: Neuroendocrine prostate cancer
TF: Transcription factor
