# Supplementary material for: A Hierarchy of Luminal Transcription Factors Defines AR Cistrome and Is Lost in Neuroendocrine Prostate Cancer
Source: Cancer Heterog Plast. Author manuscript; Available in PMC 2026 Feb 27. (PMC12945349; doi:10.47248/chp2502020008)
Supplement: supplementary material — Figure S1: FOXA1 reprograms the binding of AR, GATA2, and HOXB13 to many new binding sites that are enriched for their own motifs. Figure S2: NE markers have increased expression and variable methylation over NET. [file NIHMS2141256-supplement-supplementary_material.pdf]

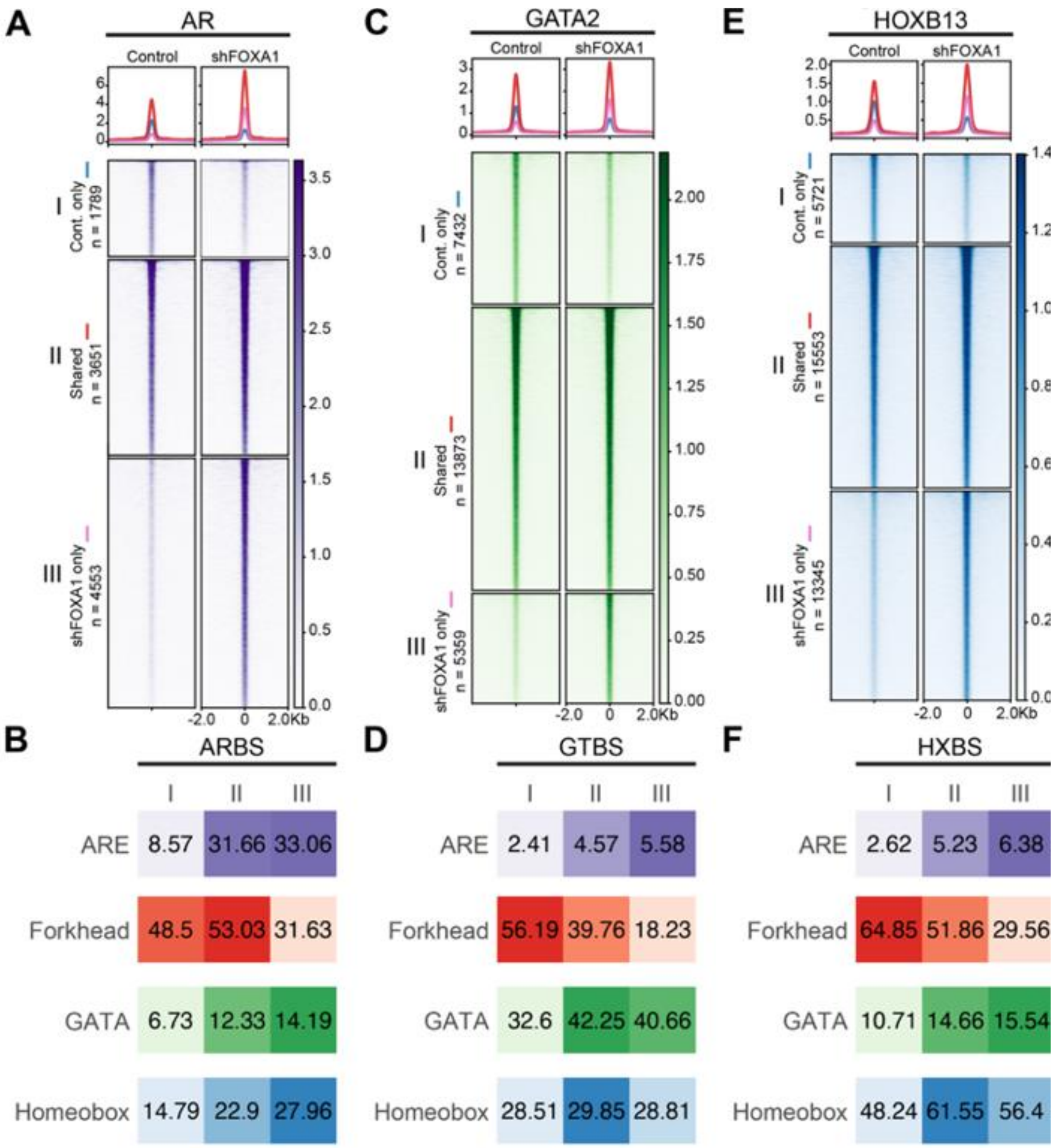

Figure S1. FOXA1 reprograms the binding of AR, GATA2 and HOXB13 to many new binding sites that are enriched for their own motifs.

A, C, E. Heatmaps showing FOXA1 knockdown lead to fewer reprogrammed FOXA1, HOXB13, GATA2 sites. Each heatmap has control-only, shared- and shFOXA1-only sites. The color bar on the side indicates the scale of the enrichment intensity. The profile plot on top shows the averaged binding intensity across each set of binding sites.

B, D, F. Enrichment of each motif of each TF (B. AR, D. GATA2, F. HOXB13) in each category in the above heatmaps. I: control only, II: shared, III: knockdown only. Numbers indicate % of target sequences with motifs found.

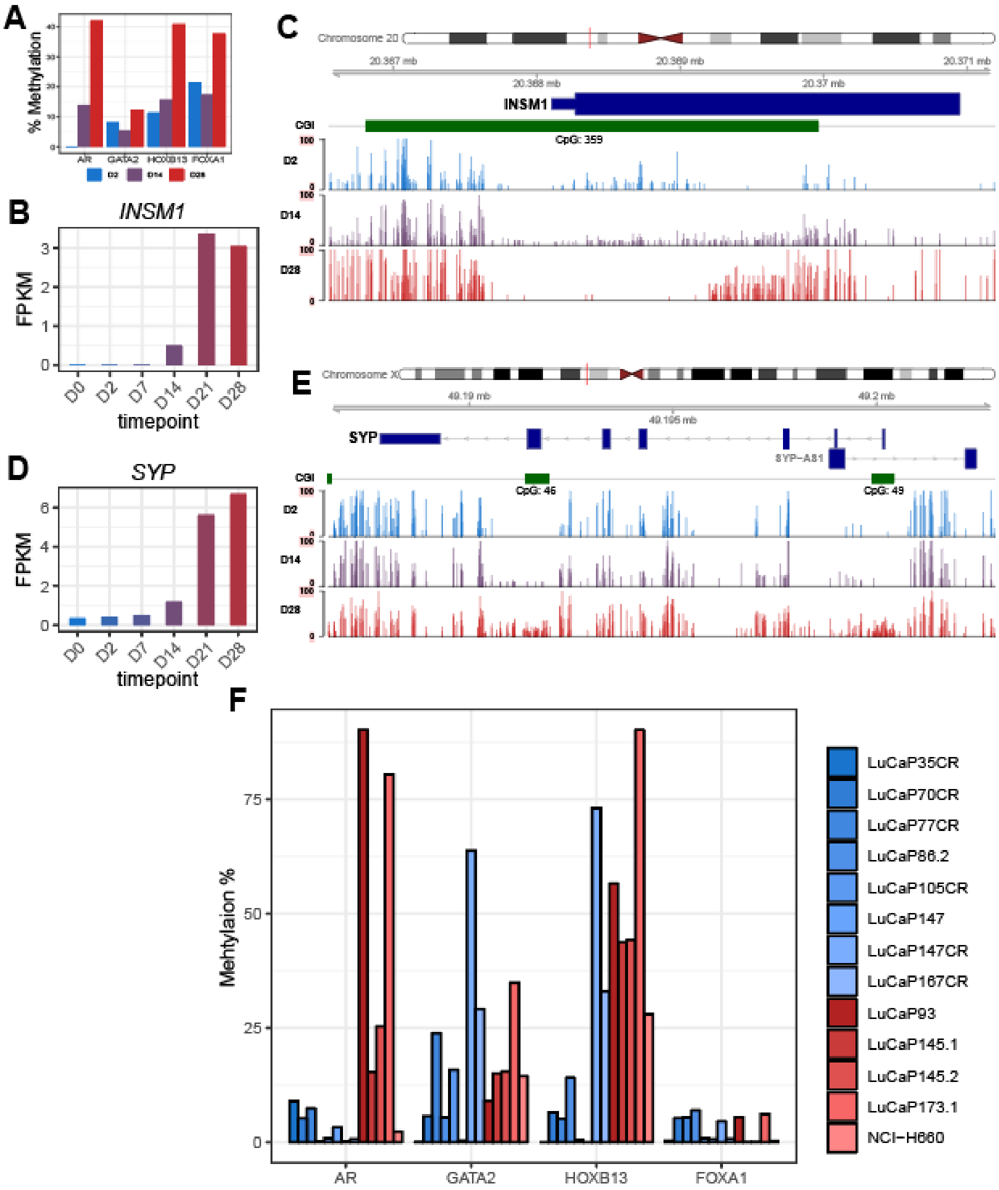

Figure S2. NE markers have increased expression and decreased methylation

A. Quantification of mean methylation levels in CpG islands of genes (*AR*: 2nd CpG:27, *GATA2*: CpG:515, *HOXB13*: CpG:21, and *FOXA1*: CpG: 115) in time-course RRMS.

B,C Gene expression levels (FPKM) of NE genes *INSM1* and *SYP* in time-course RNA-Seq data.

D,E Gviz tracks of *INSM1* and *SYP* in time-course RRMS data. CpG islands are shown in green.

F. Quantification of mean methylation levels in gene promoter CpG islands in PDXs. *AR*: 2nd CpG:27, *GATA2*: CpG:515, *HOXB13*: CpG:21, *FOXA1*: CpG: 115.
